# Initial Experience With Senhance-Assisted Laparoscopic Partial Cystectomy Using the Double Bipolar Method With 3 mm Bipolar Instruments

**DOI:** 10.7759/cureus.74074

**Published:** 2024-11-20

**Authors:** Junya Kawabata, Go Kaneko, Suguru Shirotake, Suguru Matsushita, Taku Homma, Masafumi Oyama, Isamu Koyama

**Affiliations:** 1 Uro-Oncology, Saitama Medical University International medical center, Hidaka, JPN; 2 Uro-Oncology, Saitama Medical University International Medical Center, Hidaka, JPN; 3 Diagnostic Pathology, Saitama Medical University International Medical Center, Hidaka, JPN; 4 Gastroenterological Surgery, Saitama Medical University International Medical Center, Hidaka, JPN

**Keywords:** bipolar forceps, laparoscopic partial cystectomy, maryland bipolar, partial cystectomy, robot, robot-assisted laparoscopy

## Abstract

The Senhance robotic system (Asensus Surgical, Durham, NC, USA) is an innovative platform for minimally invasive surgery. It enables surgeons to perform precise and cost-effective procedures using reusable instruments and has advanced features such as haptic feedback and eye-tracking camera control. Herein, we present the first application of the "double bipolar method" (DBM) in a Senhance-assisted laparoscopic partial cystectomy utilizing 3 mm Maryland bipolar instruments. The DBM technique allows for the simultaneous use of bipolar instruments in both hands, thereby providing exceptional control in tissue dissection and coagulation, which are critical for delicate urologic procedures such as partial cystectomy. We present a case of a 62-year-old female patient who had a 2 cm tumor located at the bladder's dome. Following comprehensive preoperative imaging and cystoscopic evaluation, the tumor was deemed suitable for resection using the Senhance system. The DBM technique enabled the precise and bloodless resection of the bladder wall. Intraoperative evaluation confirmed the complete removal of the tumor and the successful closure of the bladder defect using a barbed suture. The patient had an uncomplicated recovery and was discharged on the eighth postoperative day. The combination of Senhance's advanced features and the DBM technique with 3 mm instruments offers a significant advantage in urologic surgery, providing enhanced precision, cost-efficiency, and improved cosmetic outcomes. The DBM technique in conjunction with the Senhance system represents a promising approach for bladder-sparing surgeries, with the potential for widespread adoption in clinical practice.

## Introduction

The Senhance robotic system (Asensus Surgical, Morrisville, NC, USA), initially introduced as the TELELAP Alf-X in 2012, represents a significant advancement in the field of telesurgery [1]. The system is designed to assist with conventional laparoscopic surgery, differing from the widely used da Vinci system (Intuitive Surgical, Sunnyvale, CA, USA). The platform is equipped with up to four robotic arms, which are operated by a surgeon situated at a console with laparoscopic handles [2].

The Senhance system incorporates an eye-tracking camera control and haptic feedback, which provide the surgeon with tactile sensations, which enhance the surgeon’s ability to perform the procedure. Moreover, surgical procedures may be conducted in a comfortable seated position, thereby reducing the strain on the cervical and lumbar regions of the spine. In contrast to the da Vinci system, most instruments in the Senhance system are reusable without time limitation, which markedly reduces maintenance costs. The system received regulatory approval in Europe in 2014 and the United States in 2017 for use in general surgery, gynecology, urology, and thoracic surgery. In Japan, the device received regulatory approval in 2019 for 98 laparoscopic surgical procedures. Subsequently, in 2022, its indications were expanded to encompass additional thoracic and laparoscopic procedures.

In the field of urology, the Senhance system has been shown to possess versatility and efficacy in a diverse range of procedures [3-10]. As previously reported, the Senhance system has been shown to be a valuable tool in laparoscopic radical nephrectomy for renal cell carcinoma [7]. Subsequently, our institution has employed the Senhance system for laparoscopic adrenalectomy for adrenal tumors and laparoscopic nephroureterectomy for upper tract urothelial carcinoma. Recently, the system’s instrument range has been expanded to include 3 mm instruments. We previously reported the utility of a 3 mm Maryland bipolar instrument in laparoscopic renal surgery [11]. The instrument proved useful in various situations, including tissue grasping, blunt dissection, incision, and coagulation.

Partial cystectomy is a surgical procedure employed in bladder-sparing surgery for the treatment of bladder cancer, benign tumors like leiomyomas and hemangiomas, and diseases related to urachal remnant. The efficacy of robot-assisted partial cystectomy with the da Vinci system has been previously reported [12-15]. The Japanese insurance system does not provide coverage for robot-assisted partial cystectomy using the da Vinci system; however, partial cystectomy using the Senhance system is eligible for coverage. In the present case, a Senhance-assisted laparoscopic partial cystectomy was performed using the "double bipolar method" (DBM), which involves operating a 3 mm bipolar instrument with both hands. To the best of our knowledge, this is the first report of the efficacy of this procedure.

## Case presentation

**Figure 1:**
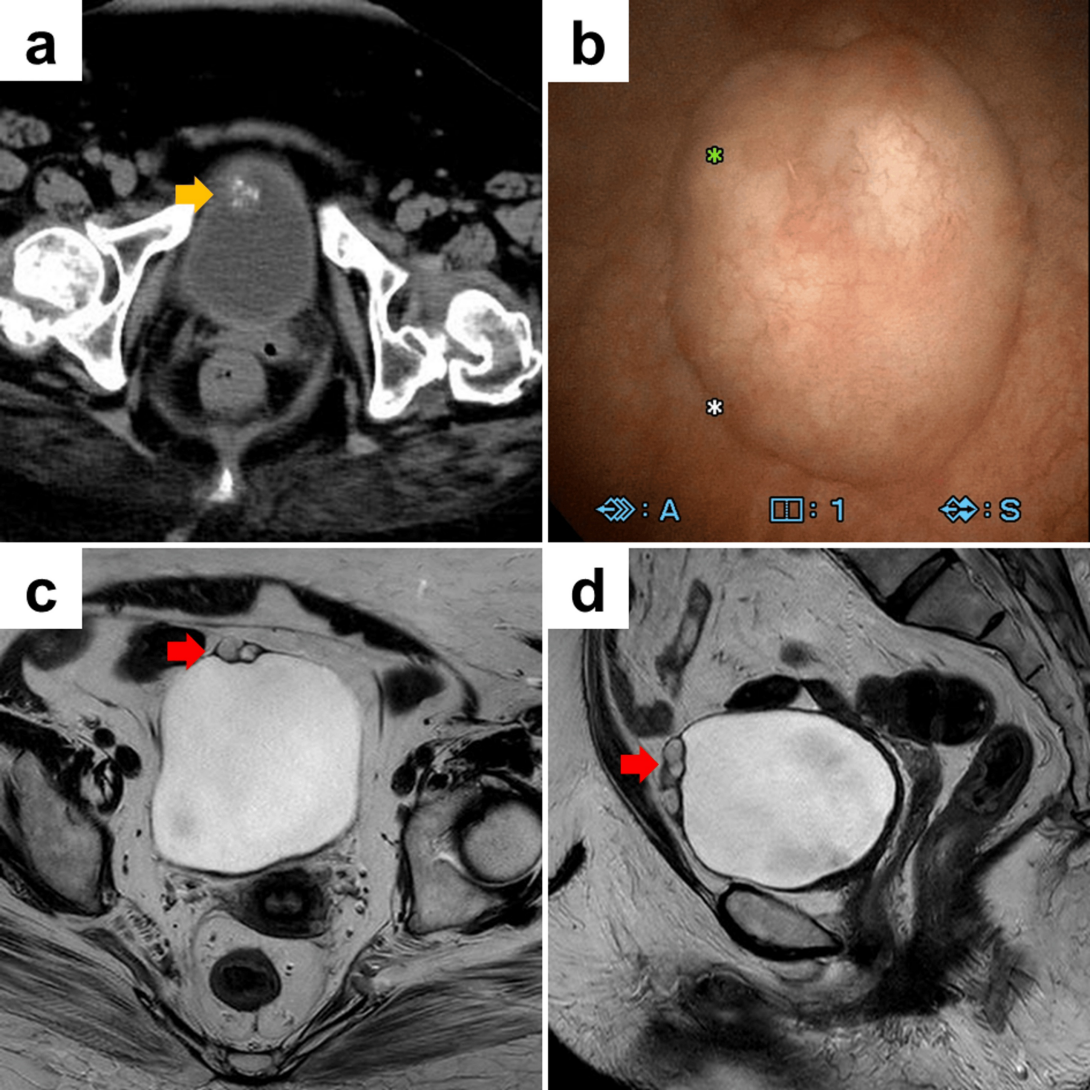
Imaging findings of the bladder tumor (a) Computed tomography (CT) shows the presence of a 2 cm-sized bladder tumor with calcification at the dome of the bladder. The yellow arrow indicates the presence of a bladder tumor. (b) Cystoscopy shows the presence of a bladder tumor at the dome. The mucosal surface of the tumor was observed to be unremarkable. (c) and (d) T2-weighted magnetic resonance imaging shows the presence of a 2 cm-sized multifocal cystic lesion in the anterior wall of the dome of the bladder (c: axial section; d: coronal section).

A 62-year-old female (body mass index: 40.8 kg/m^2^) with a history of gallstones and cerebral palsy, a side effect of the polio vaccine, was identified as having a 2 cm-sized bladder tumor with calcification at the dome of the bladder (Figure 1a) by computed tomography, which was performed for the purpose of investigating the cause of hypertension. She was subsequently referred to our hospital for further examination. Cystoscopy revealed the presence of a tumor at the dome of the bladder (Figure 1b). The mucosal surface of the tumor was observed to be unremarkable. Urine cytology revealed the absence of malignant cells, and a qualitative examination was conducted using magnetic resonance imaging. A 2 cm-sized multifocal cyst was identified in the anterior wall, from the dome of the bladder (Figure 1c-1d). No evidence of a urachal remnant was observed. In light of the patient's expressed desire to receive resection, a decision was made to perform a Senhance-assisted laparoscopic partial cystectomy.

Surgical technique

**Figure 2:**
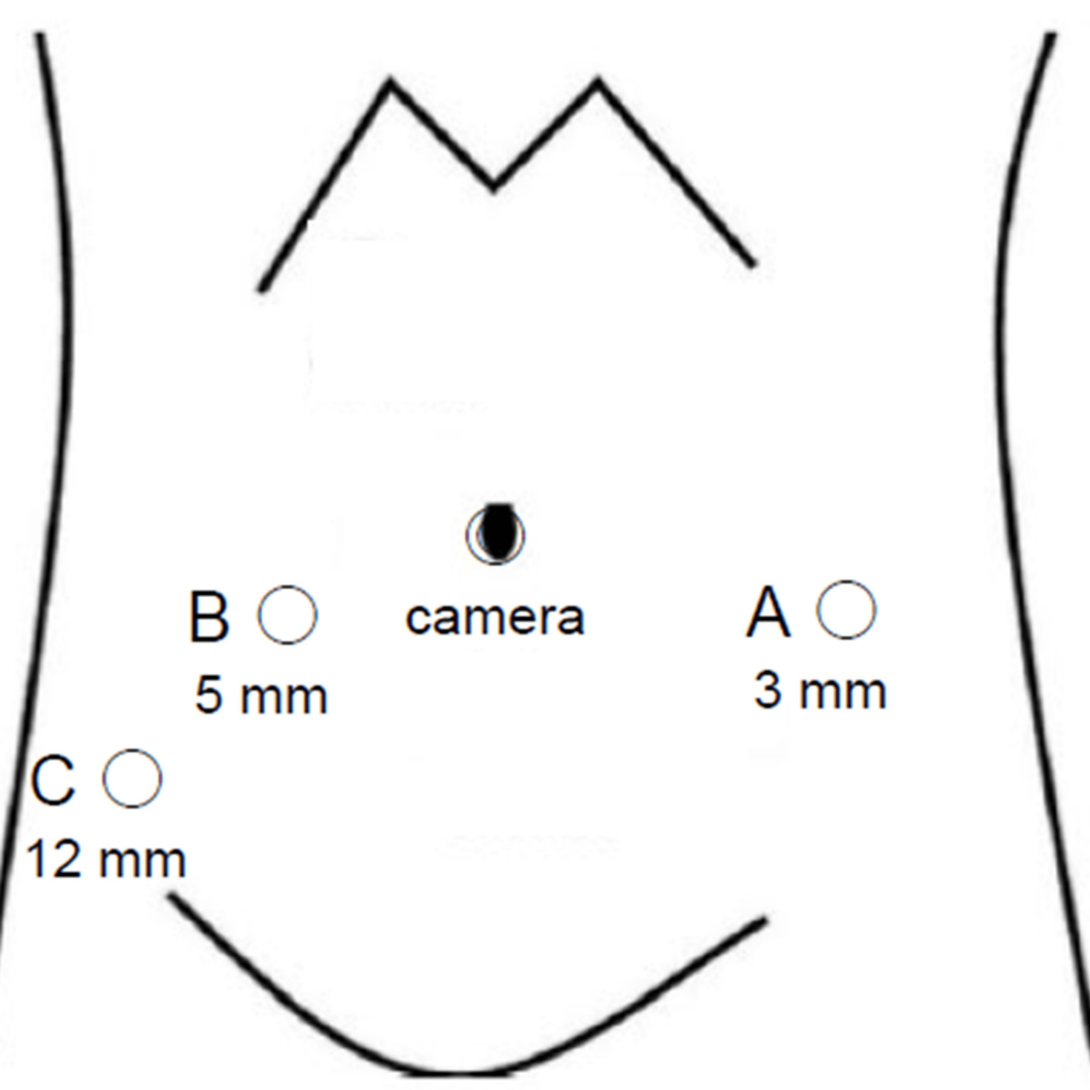
Trocar position in laparoscopic partial cystectomy using the Senhance system The camera port was placed at the umbilicus. A 3 mm trocar was inserted at a distance of 8 cm from the umbilicus, in alignment with the line connecting the umbilicus and the left superior anterior iliac spine (A). A 5 mm trocar was inserted at a distance of 8 cm from the umbilicus, in alignment with the line connecting the umbilicus and the right superior anterior iliac spine (B). A 12 mm trocar for the assistant was inserted in close proximity to the right superior anterior iliac spine (C).

**Figure 3:**
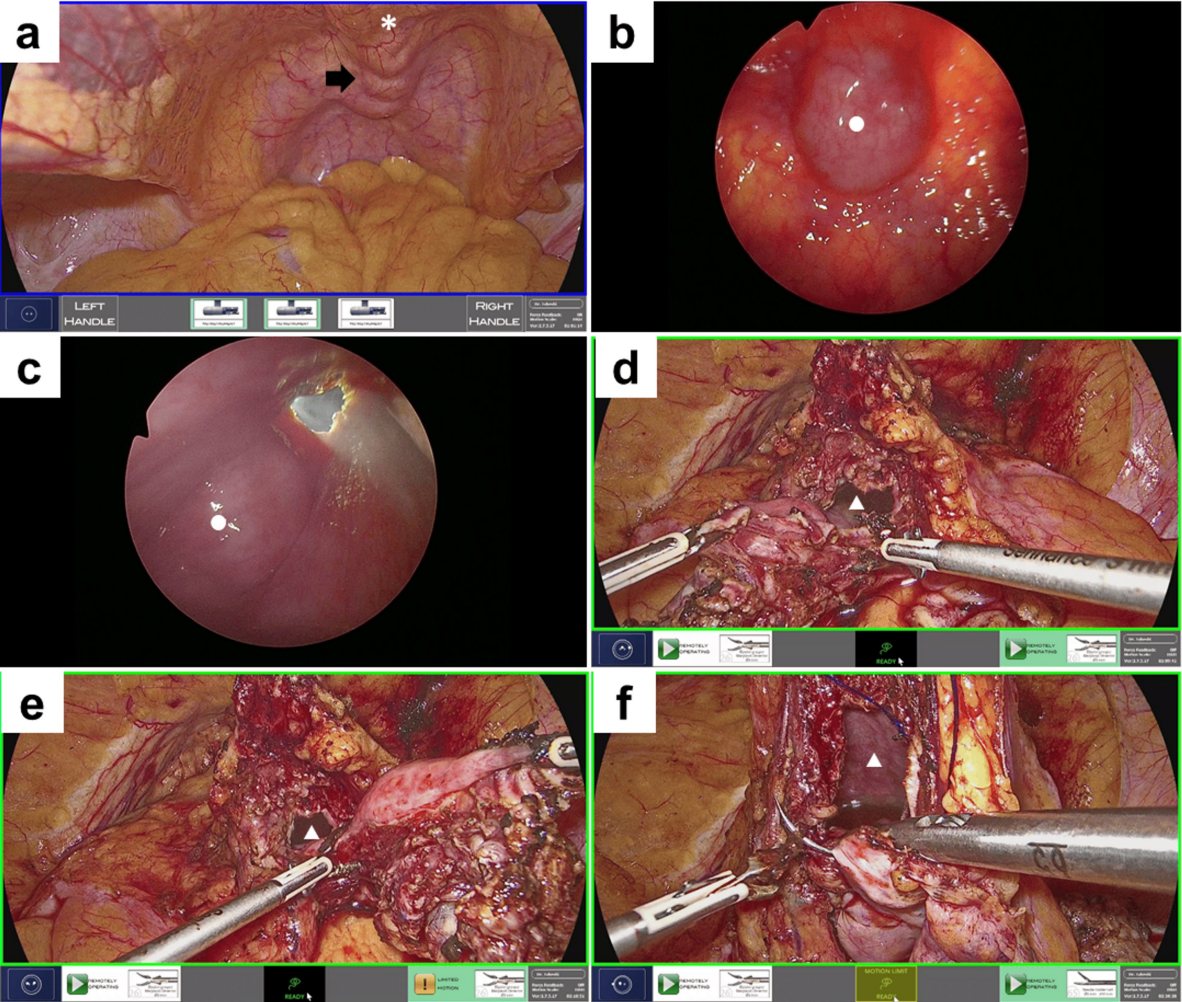
Intraoperative findings (a) The region in close proximity to the bladder in the median umbilical ligament exhibited a dilated appearance, which is indicative of the presence of a tumor (black arrow). (b and c) A cystoscopic examination was conducted to ascertain the extent of resection and its adequacy when the bladder wall was removed from the abdominal cavity. (d and e) The bladder wall was incised using a 3 mm bipolar instrument in bipolar cut mode. The 3 mm bipolar instruments were grasped bilaterally, with either instrument employed to create the incision, depending on the area to be resected. (f) The bladder defect was closed with a 3-0 spiral polydioxanone barbed suture using 5 mm needle holder. *, ○, and △ indicate the median umbilical ligament, the tumor, and a bladder defect, respectively.

The patient was placed in a lithotomy position with the head lowered by 25 degrees under general anesthesia, and a camera port was inserted at the umbilicus. Once the pneumoperitoneum had been established, three trocars were inserted in alignment (Figure 2). Ports A and B were placed at a distance of 8 cm from the umbilicus, in alignment with the line connecting the umbilicus and the superior anterior iliac spine. A 12-mm trocar for the assistant was placed in close proximity to the right superior anterior iliac spine (Port C). A 10-mm rigid laparoscope (Karl Storz) was inserted through the camera port, and the median umbilical ligament was observed to be dilated in proximity to the bladder (Figure 3a). The Retzius space was developed using a routine laparoscopic technique, employing a vessel-sealing device (Enseal, Ethicon, Inc., Bridgewater, NJ, USA) and a straight laparoscopic electrode (Endopath, Ethicon, Inc.). A 10-mm rigid laparoscope (Karl Storz SE & Co., Tuttlingen, Germany) and two 3-mm Maryland bipolar instruments were inserted via the camera port, a 3 mm trocar (port A in Figure 2), and a 5 mm trocar (port B in Figure 2), respectively, and connected to the Senhance system. The 3 mm Maryland bipolar instruments were each connected to the VIO 3 electrosurgical unit (Erbe, Dwaraka, Delhi, India). The settings were configured to Autocut mode at 5.5 W for incision and Soft Coag mode at 6 W for coagulation. A cystoscope was inserted into the bladder, which was then distended with 200 cc of air. The bipolar cut mode of the 3 mm Maryland bipolar forceps was employed to excise the peritoneum and the adipose tissue surrounding the bladder. The resection site was also observed via cystoscopy and evaluated as needed to ascertain the adequacy of the resection (Figure 3b-3c). The bladder wall was incised in bipolar cut mode using a 3 mm bipolar instrument, resulting in a bloodless procedure that permitted the performance of a precise resection. The 3 mm bipolar instruments were grasped bilaterally, with either instrument utilized to create the incision contingent upon the area to be resected (Figure 3d-3e). The bladder defect was closed with a 3-0 spiral polydioxanone barbed suture (STRATAFIX; Ethicon, Somerville, NJ) using a 5 mm needle holder (Figure 3f).

Peri- and postoperative results

**Figure 4:**
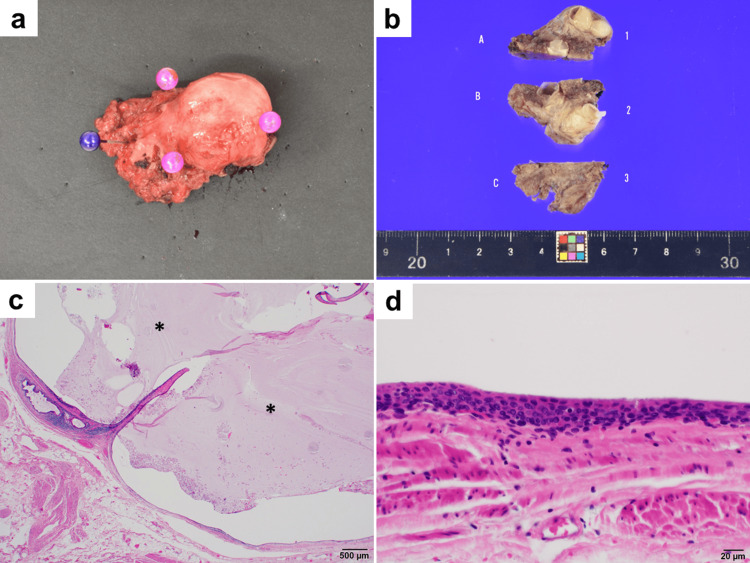
Macroscopic and microscopic findings of the excised specimen (a) A mass with a smooth surface and a maximum diameter of 25 mm was resected with partial attachment of normal bladder wall. (b) The divided plane of the cyst. The cyst was multifocal and contained a white mucus component. (c and d) Histological examination revealed that the cyst was coated with a poorly atypical multilayered epithelial component and contained an internal mucus component (*) (hematoxylin and eosin staining).

The procedure was successfully completed without conversion to conventional laparoscopic partial cystectomy. The console time and estimated blood loss were 79 minutes and 5 ml, respectively. The postoperative course was uneventful. On the seventh postoperative day, we conducted a cystogram to confirm the absence of urine leakage. Subsequently, the urethral catheter was removed. On the eighth postoperative day, the hospital discharged the patient after confirming her normal urination. The excised specimen was a multifocal cyst, measuring 25 mm × 20 mm, with a smooth surface and an internal white mucus component in the divided plane (Figure 4a-4b). Microscopic examination revealed that the cyst was covered by a poorly atypical epithelial component with an internal mucus component (Figure 4c-4d). No malignant findings were identified; consequently, the diagnosis was made as a urachal cyst.

## Discussion

We described our initial experience with Senhance-assisted laparoscopic partial cystectomy using the DBM with 3 mm Maryland bipolar instruments. To accurately dissect the tissue while minimizing bleeding, 3 mm Maryland bipolar instruments were employed in both cutting and coagulation modes during bladder wall resection. Furthermore, the surgeon was able to employ the 3 mm Maryland bipolar instrument in both hands to select the appropriate forceps on either side, based on the resection site.

Partial cystectomy is a surgical procedure performed for a range of medical conditions, including those related to the urachal remnant. The efficacy of robot-assisted partial bladder resection using the da Vinci system has been demonstrated [12-15]. However, the forceps of the da Vinci System have a limited number of uses, resulting in significant expense. Furthermore, the relatively large diameter of the trocar, which is typically 8 mm or more, presents a cosmetic challenge. The forceps of the Senhance system are designed to be repeatable, and the use of 3 mm and 5 mm forceps allows the surgeon to perform the procedure with 3 mm or 5 mm trocars, respectively. In the present case, Senhance-assisted laparoscopic partial cystectomy was chosen for its economic and cosmetic advantages.

In recent years, in Japan, DBM (da Vinci robotic system) has become widespread in the fields of general surgery and gynecology. DBM is a surgical technique in which bipolar instruments are operated with both hands, and the technique has favorable surgical outcomes [16-19]. In our previous report, the utility of 3 mm Maryland bipolar instruments in Senhance-assisted laparoscopic renal surgery was demonstrated, confirming that these instruments are capable of accurately and precisely grasping the membrane [11]. When utilized in the cutting and coagulation mode, the instrument exhibited the capacity to accurately dissect the perinephric region while concurrently preventing hemorrhage. In light of these observations, we considered the potential utility of DBM in urologic laparoscopic surgery using the Senhance system. This was the inaugural Senhance-assisted laparoscopic procedure performed with the DBM technique. The capacity to grasp, incise, and coagulate tissue with both hands ensured a highly comfortable procedure.

The bladder defect was closed with two layers of continuous sutures using a 5 mm needle holder. Although it has been reported that accurate suturing is possible using the Senhance system in vesicourethral anastomosis in prostatectomy [3-6], this was our first experience with suturing using the Senhance system. The Senhance system allows for straightforward suturing in a three-dimensional magnified field of view without any evidence of hand tremor. This facilitates the successful, watertight closure of bladder defects with precise sutures.

## Conclusions

The utilization of the DBM technique, employing a 3 mm Maryland bipolar instrument in the Senhance system, facilitated the precise resection of the bladder wall without bleeding. The Senhance system utilizes straight forceps, which may be perceived as a disadvantage for continuous suturing of bladder defects when compared to multidegree-of-freedom forceps, such as those used in the da Vinci system. Nevertheless, we were able to perform continuous suturing of the bladder defect without encountering any significant difficulties. We hope that this technique will become a widely utilized approach in partial cystectomy.
